# mTORC1 activation is not sufficient to suppress hepatic PPARα signaling or ketogenesis

**DOI:** 10.1016/j.jbc.2021.100884

**Published:** 2021-06-17

**Authors:** Ebru S. Selen, Michael J. Wolfgang

**Affiliations:** 1 Department of Biological Chemistry, The Johns Hopkins University School of Medicine, Baltimore, Maryland, USA; 2 Department of Pharmacology and Molecular Sciences, The Johns Hopkins University School of Medicine, Baltimore, Maryland, USA

**Keywords:** metabolism, fatty acid oxidation, carnitine palmitoyltransferase 2 (Cpt2), peroxisome proliferator-activated receptor alpha (PPARα), mTOR, ketogenesis, β-hydroxybutyrate (βHB), βHB, beta hydroxybutyrate, Cpt2^L−/−^, liver-specific deletion of carnitine palmitoyltransferase 2, DKO, double-KO, mTOR, mechanistic target of rapamycin, PCA, principal component analysis, PPARα, peroxisome proliferator-activated receptor alpha, TGs, triglycerides, TSC, tuberous sclerosis complex, TSC1^L−/−^, liver-specific deletion of TSC1

## Abstract

The mechanistic target of rapamycin (mTOR) is often referred to as a master regulator of the cellular metabolism that can integrate the growth factor and nutrient signaling. Fasting suppresses hepatic mTORC1 activity *via* the activity of the tuberous sclerosis complex (TSC), a negative regulator of mTORC1, to suppress anabolic metabolism. The loss of TSC1 in the liver locks the liver in a constitutively anabolic state even during fasting, which was suggested to regulate peroxisome proliferator-activated receptor alpha (PPARα) signaling and ketogenesis, but the molecular determinants of this regulation are unknown. Here, we examined if the activation of the mTORC1 complex in mice by the liver-specific deletion of TSC1 (TSC1^L−/−^) is sufficient to suppress PPARα signaling and therefore ketogenesis in the fasted state. We found that the activation of mTORC1 in the fasted state is not sufficient to repress PPARα-responsive genes or ketogenesis. Furthermore, we examined whether the activation of the anabolic program mediated by mTORC1 complex activation in the fasted state could suppress the robust catabolic programming and enhanced PPARα transcriptional response of mice with a liver-specific defect in mitochondrial long-chain fatty acid oxidation using carnitine palmitoyltransferase 2 (Cpt2^L−/−^) mice. We generated Cpt2^L−/−^; Tsc1^L−/−^ double-KO mice and showed that the activation of mTORC1 by deletion of TSC1 could not suppress the catabolic PPARα-mediated phenotype of Cpt2^L−/−^ mice. These data demonstrate that the activation of mTORC1 by the deletion of TSC1 is not sufficient to suppress a PPARα transcriptional program or ketogenesis after fasting.

The mechanistic target of rapamycin (mTOR) signaling pathway is often referred to as a master regulator of the cellular metabolism (1). mTOR exists in two independent complexes, mTorc1 and mTorc2. The activation of mTorc1 signaling by nutrients and/or growth factor signaling promotes the biosynthesis of macromolecules such as proteins and lipids required for cellular growth. As such, mTorc1 is a strong inducer of anabolic metabolism. In mammals, the switch from the fed state to the fasted state requires the suppression of mTorc1 signaling mediated by the tuberous sclerosis complex (TSC) consisting of Tsc1 and Tsc2. The TSC is a negative regulator of mTorc1 as it is a GTPase-activating protein complex for the small GTPase Ras homolog enriched in brain. Ras homolog enriched in brain directly binds to and activates mTorc1. Mutations in the TSC cause constitutive mTorc1 activity, anabolic cellular programing, and a rare genetic disease, resulting in tumor formation in multiple organ systems. Consequently, the loss of Tsc1 in the liver results in the age-dependent development of hepatocellular tumors (2). It has also been reported that the suppression of the mTorc1 complex by Tsc1 is important for the switch from anabolic to catabolic metabolism in the liver upon fasting. As such, the loss of Tsc1 and therefore inappropriate activation of the mTorc1 complex in the fasted state was shown to suppress peroxisome proliferator-activated receptor alpha (Pparα) transcriptional activity and therefore prevent hepatic ketogenesis (3).

Hepatic ketogenesis is an important adaptation during starvation (4). Upon fasting but after the depletion of hepatic glycogen, the liver produces glucose *de novo*. This gluconeogenesis is accompanied by the generation of ketone bodies from acetyl-CoA generated from abundant mitochondrial fatty acid β-oxidation (5). Ketogenesis serves two main purposes. First, it is an important mechanism to regenerate coenzyme A by utilizing acetyl units to generate the ketone bodies (acetone, acetoacetate, and beta hydroxybutyrate [βHB]). This enables the continuous oxidation of fatty acids without the need to fully oxidize acetyl-CoA in the tricarboxylic acid cycle and therefore sequestering CoA (6). Second, they serve as alternative oxidative substrates such that some tissues such as the brain can become less dependent on glucose oxidation (7, 8). The transcriptional shift in the liver to facilitate ketogenesis is largely mediated by Pparα.

Pparα is a nuclear hormone receptor that is activated by lipid ligands in the liver during fasting. Pparα drives the expression of genes in ketogenesis and fatty acid oxidation. The loss of hepatic fatty acid oxidation genes results in an increased expression of hepatic fatty acid catabolic gene expression as the mice attempt to compensate for a defect in the pathway (9, 10). The liver-specific deletion of carnitine palmitoyltransferase 2 (Cpt2^L−/−^) mice, an obligate enzyme in mitochondrial long-chain fatty acid β-oxidation, results in a robust increase in a procatabolic fasting-induced Pparα transcriptional program (5, 11, 12). This is likely mediated by the increased availability of lipids that can ligand and induce Pparα-dependent transcription. As mTorc1 is an inducer of an anabolic program and putative master regulator of cellular metabolism, we asked if the activation of mTorc1 by the deletion of its negative regulator Tsc1 could suppress the catabolic programing of Cpt2^L−/−^ mice in the fasted state.

Here, we examined if the activation of the mTorc1 complex by the deletion of Tsc1 in the liver was sufficient to suppress Pparα signaling and therefore ketogenesis in the fasted state. We found that although the activation of mTorc1 in the fasted state had a modest impact on Pparα-responsive genes, it was not sufficient to suppress ketogenesis. Furthermore, we examined if the activation of the anabolic program mediated by the activation of the mTorc1 complex in the fasted state could suppress the robust catabolic programing and enhanced Pparα transcriptional response of Cpt2^L−/−^ mice. Therefore, we generated liver-specific Cpt2^L−/−^; Tsc1^L−/−^ double-KO (DKO) mice and show that the activation of mTorc1 by the deletion of Tsc1 could not suppress the catabolic phenotype of Cpt2^L−/−^ mice. These data demonstrate that the activation of mTorc1 by the deletion of Tsc1 is not sufficient to suppress a Pparα transcriptional program or ketogenesis after a fast.

## Results

### Generation of mice with a combined liver-specific loss of fatty acid oxidation and activation of mTorc1

**Figure 1:**
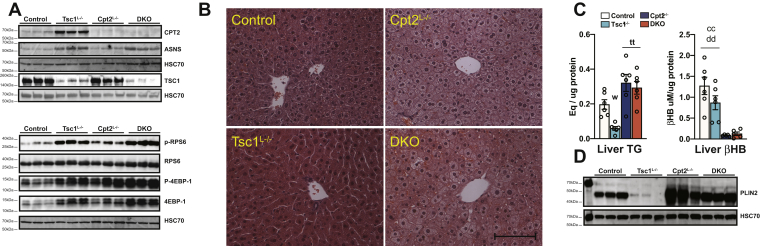
**Generation and characterization of mice with liver-specific single- and double-KOs of Cpt2 and Tsc1.***A*, Western blot for Cpt2, Tsc1, Hsc70, and mTOR’s downstream substrates in fasted liver extracts from control, Tsc1^L−/−^ (Tsc1), Cpt2^L−/−^ (Cpt2), and Tsc1Cpt2^L−/−^ (DKO) mice (n = 3). *B*, hematoxylin and eosin staining of livers from control, Tsc1^L−/−^, Cpt2^L−/−^ and Tsc1Cpt2^L−/−^ mice. The scale bar represents 100 μM. *C*, liver triglyceride and beta hydroxybutyrate (βHB) content of fasted control, Tsc1^L−/−^, Cpt2^L−/−^, and Tsc1Cpt2^L−/−^ mice (n = 6). *D*, Western blot for Plin2 and Hsc70 in fasted liver extracts of control, Tsc1^L−/−^, Cpt2^L−/−^, and Tsc1Cpt2^L−/−^ mice. One-way ANOVA followed by Tukey’s multiple comparison test was performed where appropriate to detect significance between genotypes. The *single letter* denotes *p* < 0.05, and *double letters* denote *p* < 0.01. Letters w (for control), t (for Tsc1), c (for Cpt2), and d (for DKO) represent significance between the genotypes. Data are represented as the mean ± SEM. Cpt2^L−/−^, liver-specific deletion of carnitine palmitoyltransferase 2; DKO, double-KO; TSC1^L−/−^, liver-specific deletion of TSC1.

Previously, we showed that the loss of hepatic fatty acid oxidation in the Cpt2^L−/−^ mice resulted in a robust increase in a procatabolic fasting-induced Pparα-dependent transcriptional program (5, 11, 12). Alternatively, it has been previously reported that activating mTorc1 signaling in the liver by removing its negative regulator Tsc1 (Tsc1^L−/−^ mice) was required for fasting-induced Pparα signaling and ketogenesis (3). Therefore, we examined whether activating mTorc1 and clamping the fasted liver in an mTorc1-dependent anabolic state could inhibit the dramatic Pparα transcriptional response seen in mice with defective hepatic fatty acid oxidation (Cpt2^L−/−^ mice). To accomplish this, we generated mice with liver-specific KOs of Cpt2, Tsc1 and Cpt2;Tsc1 DKO mice. First, we confirmed the loss of Cpt2 and Tsc1 in their respective models by Western blotting (Fig. 1*A*). Surprisingly, the loss of Tsc1 was associated with a marked increase in Cpt2 protein, further suggesting an interaction between the two pathways. To investigate the activation of the mTorc1 pathway by the loss of Tsc1, we examined the phosphorylation of canonical mTorc1 targets such as RPS6 and 4EBP-1. These targets are phosphorylated in fed livers by the mTorc1 complex and dephosphorylated in fasted livers. The loss of Tsc1 removes the negative regulation on mTorc1 and maintains RPS6 and 4EBP-1 phosphorylation even within the fasted state, as seen in both Tsc1^L−/−^ and DKO livers (Fig. 1*A*). In addition, asparagine synthetase, a target of mTorc1-mediated activation of ATF4, is induced in the livers of Tsc1^L−/−^ and DKO mice.

The loss of Cpt2 and Tsc1 has seemingly opposite effects on hepatic triglyceride content. The loss of Cpt2 and therefore fatty acid oxidation results in an increase in fasting-induced hepatic triglyceride accumulation (5). The loss of Tsc1 results in livers with a decrease in hepatic triglyceride accumulation (13, 14). Consistent with these known roles, fasting resulted in pale lipid-laden livers in Cpt2^L−/−^ mice and dark red lipid-poor livers in Tsc1^L−/−^ mice. However, DKO livers were pale and lipid laden, suggesting that the loss of Tsc1 was not able to suppress triglyceride accumulation in Cpt2^L−/−^ mice (Fig. 1*B*). Quantification of liver triglycerides (TGs) showed the same pattern of suppression of TGs in Tsc1^L−/−^ and increases in Cpt2^L−/−^ and DKO mice (Fig. 1*C*). We also measured the concentration of fasting liver βHB by LC/MS, and contrary to previous reports (3, 15), Tsc1^L−/−^ liver had an equal concentrations of βHB to control livers, whereas Cpt2^L−/−^ and DKO liver exhibited a marked reduction (Fig. 1*C*). In addition, we performed Western blotting for Plin2, a lipid droplet protein that is stabilized by association with lipid droplets. Consistent with the liver histology and TG concentration, Plin2 was suppressed in Tsc1^L−/−^ mice and induced in Cpt2^L−/−^ and DKO mice (Fig. 1*D*). These results are consistent with the known roles of Tsc1 and Cpt2 in the fasted liver and demonstrate the generation of a novel mouse model of activated hepatic mTorc1 signaling in the setting of impaired hepatic fatty acid catabolism.

### Activating mTorc1 is not sufficient to suppress fasting-induced ketogenesis

**Figure 2:**
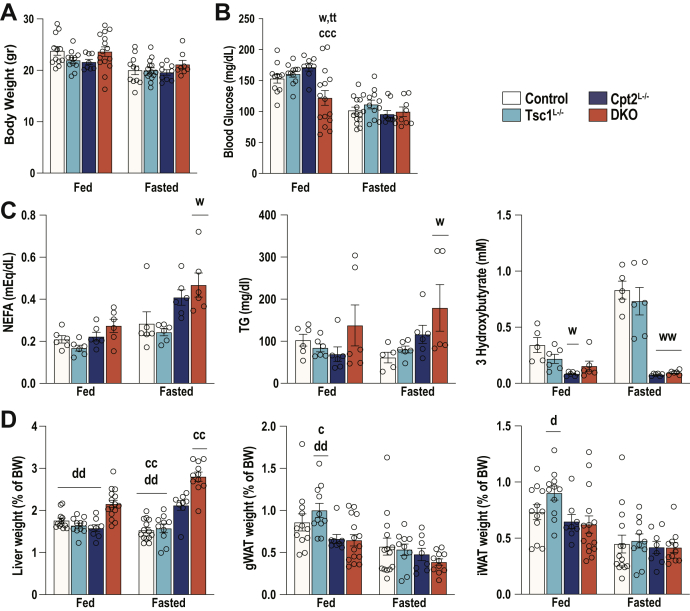
**Physiological profiling of fed and 24-h-fasted mice with liver-specific single and double KOs of Cpt2 and Tsc1.***A*, body weights of male mice under chow diet (n = 8–15). *B*, blood glucose levels of male mice measured at the time of sacrifice (n = 8–15). *C*, serum metabolites of male mice (n = 5–6). *D*, weights of the liver and gonadal white adipose (gWAT) and inguinal white adipose (iWAT) tissues, represented as the percentage of the body weight in male mice (n = 8–15). One-way ANOVA followed by Tukey’s multiple comparison test was performed where appropriate to detect significance between genotypes. The *single letter* denotes *p* < 0.05, and *double letters* denote *p* < 0.01. Letters w (for control), t (for Tsc1), c (for Cpt2), and d (for DKO) represent significance between the genotypes. Data are represented as the mean ± SEM. Cpt2, carnitine palmitoyltransferase 2; DKO, double-KO; Tsc1, tuberous sclerosis complex 1.

**Figure 3:**
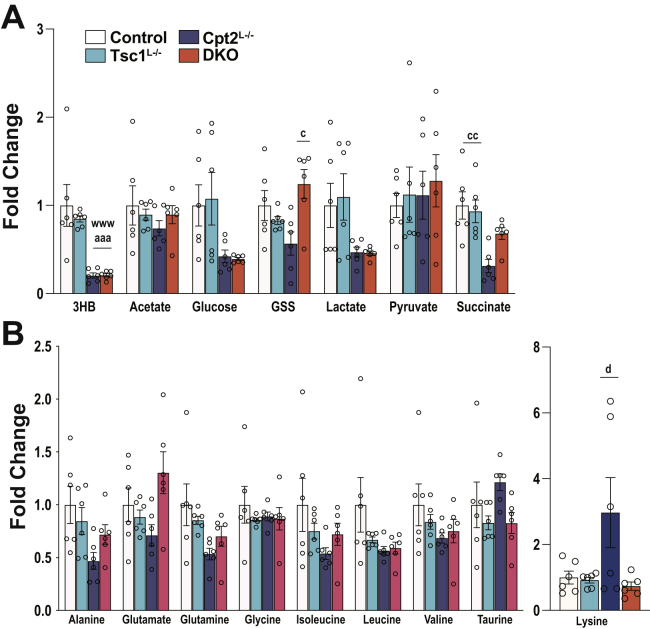
**NMR metabolomics of liver reveals a requirement for fatty acid oxidation but not mTorc1 in ketogenesis.***A*, fold differences of energy metabolism intermediates in control, Tsc1^L−/−^, Cpt2^L−/−^, and Tsc1Cpt2^L−/−^ livers (n = 6). *B*, fold differences of physiological amino acids in control, Tsc1^L−/−^, Cpt2^L−/−^, and Tsc1Cpt2^L−/−^ livers (n = 6). One-way ANOVA followed by Tukey’s multiple comparison test was performed where appropriate to detect significance between genotypes. The *single letter* denotes *p* < 0.05, and *double letters* denote *p* < 0.01. Letters w (for control), t (for Tsc1), c (for Cpt2), and d (for DKO) represent significance between the genotypes. Data are represented as the mean ± SEM. Cpt2^L−/−^, liver-specific deletion of carnitine palmitoyltransferase 2; DKO, double-KO; TSC1^L−/−^, liver-specific deletion of TSC1.

To understand the effect of activating hepatic mTorc1 in a model of enhanced Pparα signaling, we phenotyped control, Cpt2^L−/−^, Tsc1^L−/−^, and DKO mice in the fed and 24-h-fasted state. The loss of Cpt2, Tsc1, or both did not have an effect on the body weights of 9-week-old male mice (Fig. 2*A*). Although the loss of these genes had no effect on fasting blood glucose concentrations, DKO mice exhibited increased circulating NEFA and TGs as expected (Fig. 2*C*). It has been previously reported that mice with a liver-specific loss of Tsc1 exhibit suppressed ketone body production using the identical KO strategy used here (3). Therefore, we were surprised to see that the loss of Tsc1 had no effect on the generation of serum βHB in the fed or the fasted state in comparison with control mice. Cpt2^L−/−^ and DKO exhibited a marked suppression of serum βHB because of their inability to oxidize fatty acids in the liver (Fig. 2*C*). Liver weight increased in the KO mice as expected with fasted DKO mice having enlarged livers without effecting adiposity (Fig. 2*D*). To further understand the role of Tsc1 in the generation of ketone bodies, we directly profiled the livers of control, Cpt2^L−/−^, Tsc1^L−/−^, and DKO mice in the fasted state by ^1^H-NMR metabolomics. Again, the Tsc1^L−/−^ livers exhibited no change in the βHB concentration, whereas Cpt2^L−/−^ and DKO mice had a marked suppression of βHB (Fig. 3*A*). Other metabolites profiled were not consistently altered by the absence of Tsc1 save the amino acid lysine which was significantly suppressed in the DKO (Fig. 3*B*). These data show that the activation of mTorc1 is not sufficient to suppress hepatic ketogenesis.

**Figure 4:**
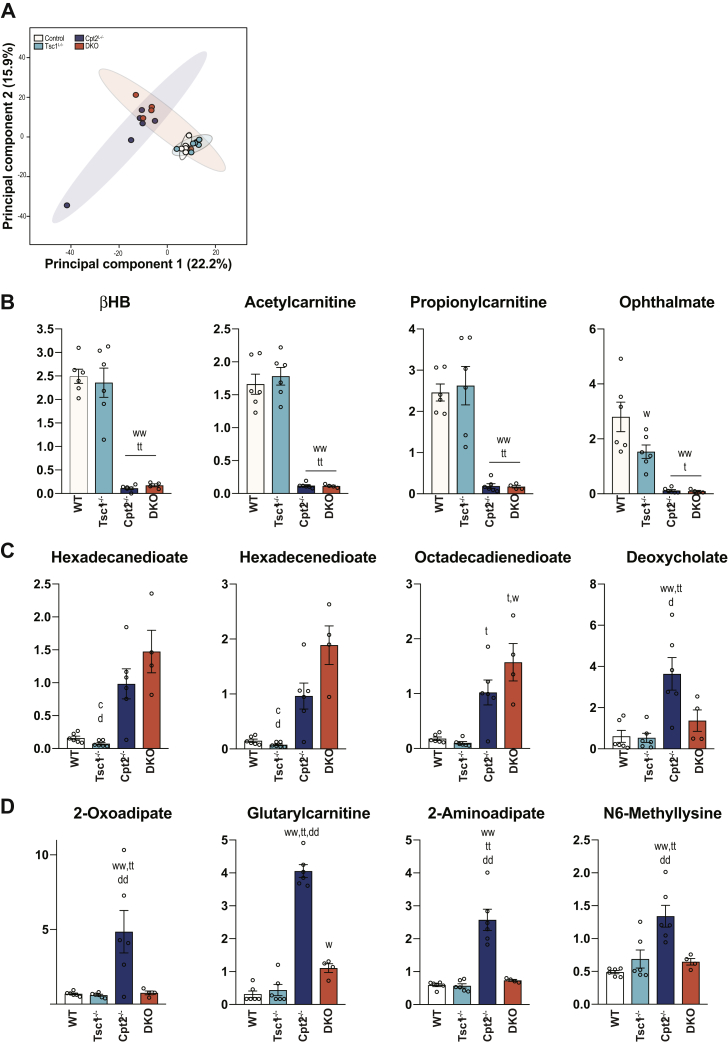
**Global unbiased metabolome profiling of fasted mice demonstrates unique contributions of fatty acid oxidation and mTorc1.***A*, principal component analysis of liver metabolome data (n = 6). *B* and *C*, metabolite levels that are dominated by loss of Cpt2 in the livers (n = 6). *D*, metabolite levels that are dominated by loss of Tsc1 in the livers (n = 6). One-way ANOVA followed by Tukey’s multiple comparison test was performed where appropriate to detect significance between genotypes. The *single letter* denotes *p* < 0.05, and *double letters* denotes *p* < 0.01. Letters w (for control), t (for Tsc1), c (for Cpt2), and d (for DKO) represent significance between the genotypes. Data are represented as the mean ± SEM. Cpt2, carnitine palmitoyltransferase 2; DKO, double-KO; Tsc1, tuberous sclerosis complex 1.

To expand upon the metabolites profiled in by ^1^H-NMR, we used unbiased discovery-based metabolomics to profile the liver metabolome of control, Cpt2^L−/−^, Tsc1^L−/−^, and DKO mice in the fasted state. Principal component analysis (PCA) demonstrated that Tsc1^L−/−^ and control mice clustered and Cpt2^L−/−^ and DKO mice clustered (Fig. 4*A*). Again, utilizing a fourth independent measure, Tsc1^L−/−^ livers did not exhibit a suppression in βHB, whereas Cpt2^L−/−^ and DKO mice had a marked suppression in liver βHB (Fig. 4*B*). Consistent with the PCA, loss of Cpt2 dominated the metabolic changes in DKO mice (Fig. 4, *B*–*D*). The ^1^H-NMR analysis of DKO livers demonstrated a Tsc1-dependent suppression of lysine in Cpt2^L−/−^ mice. Consistent with these data, unbiased metabolomics demonstrated a suppression in lysine catabolic products such as 2-oxoadipate, glutarylcarnitine, and 2-aminoadipate in the DKO liver (Fig. 4*D*). These data confirm that Tsc1^L−/−^ mice do not have an inherent defect in ketogenesis and have limited ability to suppress the catabolic phenotype of Cpt2^L−/−^ mice.

### Activating mTorc1 is not sufficient to suppress fasting-induced Pparα signaling

**Figure 5:**
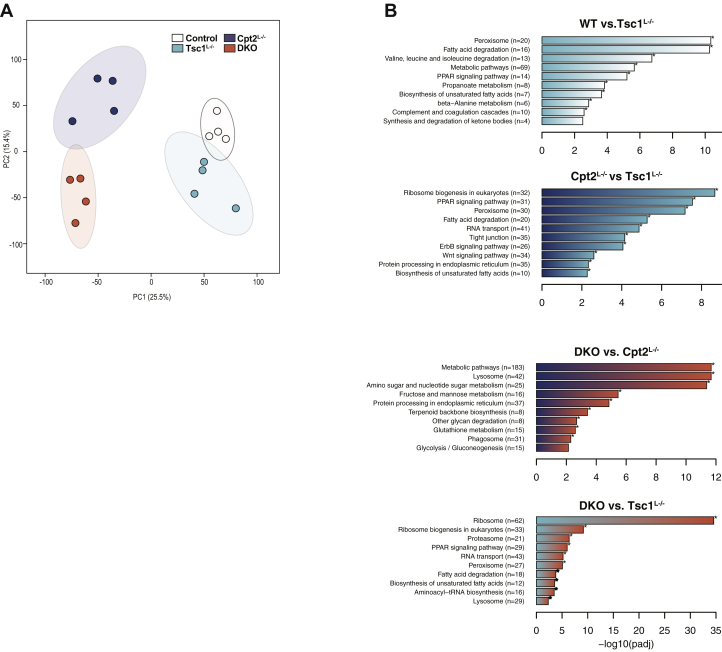
**RNAseq analysis reveals differential transcriptional regulation by activated mTorc1 and suppressed fatty acid oxidation.***A*, principal component analysis of liver RNASeq data (n = 4). *B*, pairwise Kyoto Encyclopedia of Genes and Genome analysis showed significantly altered pathways and processes in Cpt2- and/or Tsc1-deficient livers. Cpt2, carnitine palmitoyltransferase 2; mTorc1, mechanistic target of rapamycin complex 1; PC, principal component; Tsc1, tuberous sclerosis complex 1.

Previously, it had been suggested that Tsc1^L−/−^ mice exhibited defective ketogenesis because of suppression of Pparα signaling by the activated mTorc1 complex (3). To understand the effect of activating hepatic mTorc1 in a model of enhanced Pparα signaling, we performed RNA-seq on control, Cpt2^L−/−^, Tsc1^L−/−^, and DKO mice after a 24-h fast. Similar to the analysis of the metabolomic data, PCA demonstrated that Tsc1^L−/−^ and control mice clustered more closely together and Cpt2^L−/−^ and DKO mice clustered more closely together (Fig. 5*A*). Pathway analysis demonstrated that Cpt2^L−/−^ mice exhibited enhanced Pparα signaling, whereas Tsc1^L−/−^ mice exhibited suppressed Pparα signaling by the Kyoto Encyclopedia of Genes and Genome pathway analysis (Fig. 5*B*). However, the loss of Tsc1 on the Cpt2^L−/−^ background was not sufficient to suppress Pparα signaling. DKO mice do not exhibit a suppression in Pparα, suggesting that the deletion of Tsc1 is not sufficient to suppress fasting-induced Pparα in Cpt2^L−/−^ liver, a physiological model of enhanced Pparα signaling.

**Figure 6:**
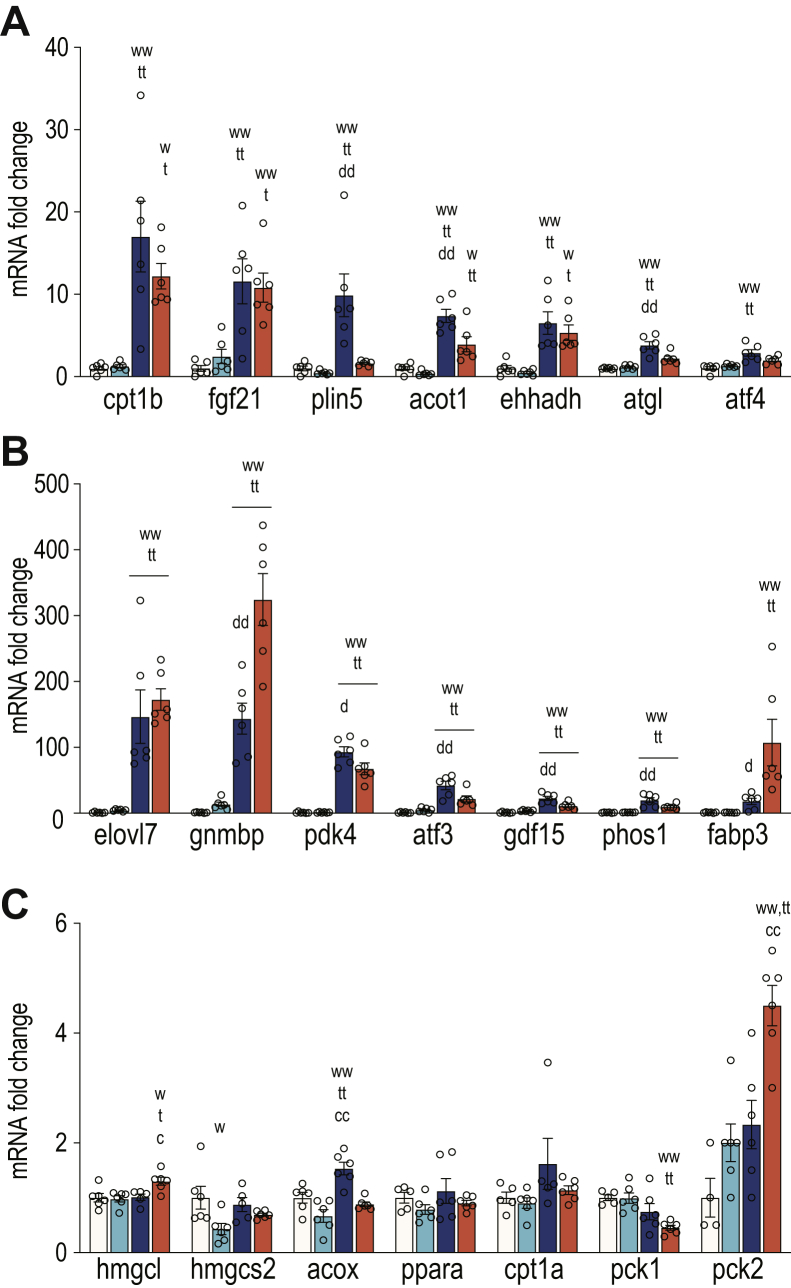
**Activating mTorc1 is not sufficient to suppress fasting-induced Pparα signaling.***A*, higher fold changes of selected Pparα target gene expression of 24-h-fasted liver (n = 6). *B*, mid-range fold changes of selected Pparα target gene expression of 24-h-fasted liver (n = 6). *C*, lower fold changes of selected Pparα target gene expression of 24-h-fasted liver (n = 6). One-way ANOVA followed by Tukey’s multiple comparison test was performed where appropriate to detect significance between genotypes. The *single letter* denotes *p* < 0.05, and *double letters* denotes *p* < 0.01. Letters w (for control), t (for Tsc1), c (for Cpt2), and d (for DKO) represent significance between the genotypes. Data are represented as the mean ± SEM. Cpt2, carnitine palmitoyltransferase 2; DKO, double-KO; mTorc1, mechanistic target of rapamycin complex 1; PPARα, peroxisome proliferator-activated receptor alpha; Tsc1, tuberous sclerosis complex 1.

We next validated the RNA-seq data by qPCR by selecting known Pparα-dependent genes. Consistent with the RNA-seq analysis, the loss of Cpt2 resulted in a robust induction of canonical Pparα target genes such as Elovl7, Fgf21, Acot1, and so forth. However, we could not demonstrate the suppression of these targets in Tsc1^L−/−^ mice. In addition, these Pparα-dependent genes could not be suppressed by the activation of mTorc1 in DKO livers as DKO livers exhibited a similar induction of these genes as Cpt2^L−/−^ livers (Fig. 6). There are several notable exceptions such as Plin5, which was suppressed in Tsc1^L−/−^ livers. These data show that the activation of mTorc1 by the deletion of Tsc1 is not sufficient to suppress the activation of Pparα transcriptional program even in mice with a physiologically elevated Pparα response.

## Discussion

The liver has a large dynamic range for fatty acid β-oxidation as it vacillates between the *de novo* synthesis of fatty acids during the carbohydrate-fed state and the oxidation of adipose-derived fatty acids during fasting or low-carbohydrate feeding. Fatty acid β-oxidation plays several important roles during fasting as it provides mitochondrial acetyl-CoA for ketogenesis and along with the abundant reducing equivalents (*e.g.*, NADH) the redirection of carbon skeletons toward gluconeogenesis (16). Given the important roles of hepatic fatty acid β-oxidation, we were surprised that mice with a loss of hepatic Cpt2 could maintain normal blood glucose but a loss of ketone body production after a 24-h fast (5, 11, 12). This is possible due to both cell autonomous and non–cell autonomous compensatory processes to remarkably maintain systemic homeostasis such as the upregulation of gluconeogenesis in the kidney (5, 17). Cpt2^L−/−^ livers sense and respond to a loss in fatty acid β-oxidation by inducing catabolic genes in the liver and peripheral tissues. This is done, at least in part, by inducing Pparα-responsive genes involved in the metabolic response to fasting and is shared with other models of impaired fatty acid oxidation (9, 10).

The robust procatabolic response of Cpt2^L−/−^ mice to fasting lies in stark contrast to Tsc1^L−/−^ mice that maintain an anabolic phenotype even after a 24-h fast because of the inability of these mice to suppress the mTorc1 complex (3). Tsc1^L−/−^ mice were reported to exhibit suppressed Pparα signaling during a 24-h fast, which resulted in a suppression in ketogenesis. Although we have also observed that Tsc1^L−/−^ mice have a small suppression in the Pparα transcriptional response after a pathway analysis of significantly changed genes in our RNA-seq data, we were unable to observe any defect in ketogenesis. Similarly, others have failed to observe a defect in ketogenesis after the deletion of Tsc1 in adult mice (18). The complete loss of Pparα results in suppressed fasting ketogenesis. However, Pparα KO mice still generate abundant ketone bodies in contrast to Cpt2^L−/−^ mice (12). Although the loss of Tsc1 can suppress a small subset of Pparα response genes, it is not sufficient to elicit a biologically meaningful effect on ketone body synthesis.

Sengupta *et al.* suggested that there is a significant interaction of mTorc1 and Pparα in an age-dependent suppression of ketogenesis (3). However, this is difficult to interpret, given that Tsc1^L−/−^ mice exhibit spontaneous hepatic tumors at 9 to 10 months, which could independently effect ketogenesis (2). A whole-body KO of the mTorc1 effector S6k1 results in increased energy expenditure (19) and S6k2 whole-body KO results in increased fasting-induced ketogenesis (20). Given the role of mTOR in other tissues such as adipocytes, it is unclear if this represents a role of mTOR specifically in hepatocytes. Some have reported that Tsc1^L−/−^ mice have an increase in Cpt1a, the rate-limiting step in mitochondrial fatty acid oxidation and therefore would be expected to increase rather than decrease ketogenesis (21). In addition, Tsc1^L−/−^ mice have been reported to exhibit an induction of Fgf21 *via* Pgc1α (22). Hepatic Fgf21 is a gene exquisitely Pparα sensitive (23, 24). This would be inconsistent with a suppressive role of mTorc1 in Pparα signaling although we were unable to observe an increase in hepatic Fgf21 in Tsc1^L−/−^ mice upon fasting.

Cpt2^L−/−^ and Tsc1^L−/−^ mice exhibit opposing liver phenotypes, highly catabolic and anabolic, respectively. While the mTOR pathway is often described as a master regulator of cellular metabolism, the genetic activation of the mTorc1 pathway by the deletion of Tsc1 did not reverse the robust catabolic phenotype of Cpt2^L−/−^ mice. In fact, DKO mice closely resemble the cellular, molecular, and metabolic phenotype of Cpt2^L−/−^ mice. That is, the loss of hepatic fatty acid oxidation largely drives the phenotype in fasting mice. This occurs in spite of mTorc1 playing an important role in regulating hepatic lipid metabolism. These results underscore the importance and predominance of hepatic fatty acid β-oxidation during fasting and starvation.

## Experimental procedures

### Animals

Control Cpt2 lox/lox and Cpt2^L−/−^ mice generated and maintained on a C57BL6 background were previously described (5). Mice were housed in ventilated racks with a 14-h/10-h light/dark cycle and fed a standard chow diet (2018SX, Teklad Global) with a room temperature of 21 °C, 50% humidity, and water provided *ad libitum*. To generate Tsc1;Cpt2 double liver-specific KO mice, we bred Tsc1 f/f mice backcrossed nine generations onto a C57BL6 background (JAX# 5680 (25)) to Cpt2 f/f mice. Albumin-Cre mice backcrossed nine generations onto a C57BL6 background were obtained from Jackson Laboratory (JAX# 3574). Control mice were Tsc1 f/f; Cpt2 f/f littermates. For fasting experiments, 9-week-old mice were food-deprived for 24 h (3 PM–3 PM). For fed studies, mice were food-deprived for 4 h (11 AM–3 PM). All procedures were performed in accordance with the NIH’s Guide for the Care and Use of Laboratory Animals and under the approval of the Johns Hopkins Medical School Animal Care and Use Committee.

### Quantitative real-time PCR

RNA was isolated from liver tissue using TRIzol reagent and was further purified using RNeasy Mini Kit (QIAGEN), as recommended by the manufacturer. The MultiScribe High-Capacity cDNA reverse transcription kit (Applied Biosystems) was used to synthesize cDNA from 1 μg/μl RNA input. Two nanograms per microliter cDNA was amplified with SsoAdvanced SYBR Green Master Mix (Bio-Rad) in the presence of selected primers. 18S and cycloA were used as housekeeping genes. Expression of genes were normalized to the average of 18S and cycloA. Data are expressed as 2ˆ^−(dCt)^. Primers were previously published (26).

### Western blot

Frozen liver tissue pieces homogenized in RIPA buffer (50 mM Tris HCl, pH 7.4, 150 mM NaCl, 1 mM EDTA, 1% Triton X-100, 0.25% deoxycholate) with PhosSTOP phosphatase inhibitor (Roche) and protease inhibitor cocktail (Roche). Homogenates were centrifuged at 4 °C for 10 min at 13,000*g*. Supernatants were transferred to a new tube, and total protein concentrations were quantified by the BCA assay (Thermo Scientific). Thirty microgram of lysate was separated by Tris-Glycine SDS-PAGE (10% and 12% polyacrylamide), followed by a transfer to PVDF membranes (immobilon). Membranes were block with 5% nonfat milk in Tris-buffered saline with Tween 20 for an hour and were incubated with primary antibodies at 1:1000 (primary antibodies: Cpt2, Thermo Pierce, PAS-122117; asparagine synthetase (Asns) Santa Cruz Biotechnology SC-365809, Tuberous Sclerosis 1 (Tsc1), Cell Signaling, #4906; mTOR substrate antibody sampler Kit, Cell signaling #9862; total S6 Ribosomal Protein (RPS6) Cell signaling, #2217, total eukaryotic translation initiation factor 4E-binding protein (4EBP-1), Cell signaling, #9452; Perilipin2 (Plin2), Sigma-Aldrich, HPA016607) overnight. Heat shock chaperone 70 (Hsc70, Santa Cruz, 7298) was used at 1:1000 as the loading control. Horse radish peroxidase (HRP)-conjugated anti-rabbit (GE Healthcare NA934V) or florescence-based (Cy3-conjugated anti-mouse or Cy5-conjugated anti-rabbit, Invitrogen) secondaries were used at 1:1000 where appropriate. Proteins were visualized with Amersham Prime enhanced chemiluminescent substrate (GE Healthcare) or epifluorescence on Alpha Innotech MultiImage III instrument.

### Histology

For liver histology, tissue was fixed in 10% neutral buffered formalin, embedded in paraffin, sectioned, and stained with hematoxylin and eosin or processed for trichrome staining (AML Laboratories Inc).

### Serum and tissue metabolites

Enzymatic and colorimetric assays were used to measure serum levels of βHB (Stanbio BHB LiquiColor Assay, EKF Diagnostics), nonesterified fatty acids, NEFA (NEFA-HR(2), Wako Diagnostics), and TG (TR0100, Sigma-Aldrich). Tissue TG levels were measured as reported previously (12). Lipid peroxidation in liver tissue was measured with Thiobarbituric Acid Reactive Substances assay (TBARS, Cayman Chemical) as directed by the manufacturer. Untargeted metabolomics from flash-frozen liver samples was performed by Metabolon Inc. Tissue 3-HB measured by LC-MS. Liver samples were homogenized in 80% methanol-water mixture, vortexed for 30 s, and centrifuged at 13,000*g* for 10 min at 4 °C. The supernatant was transferred to a new tube and placed into speed-vac overnight. The pellet was resuspended in 0.5 M sodium hydroxide overnight, and the supernatant was used to quantify the proteins for data normalization purposes. Dried samples were reconstituted in 200 μl water just before the LC-MS/MS run. Kinetex Core-shell C18 column (2.6 μm, 50 mm, 2.1 mm, Phenomenex) was used to acquire data. Mobile phases are A: water + 0.2% formic acid and B: can + 0.2% formic acid. Data were collected on Shimadzu Nexera UHPLC (Shimadzu), coupled to 4500 Triple quadruple (Ab Sciex) instrument. Total run time is 5 min, with a flow rate of 0.2 ml/min. The gradient is applied as follows; 0% B at 0 min, 5% B at 0 to 4 min, 0% B at 4.1 to 5 min. Injection volume is 2 μl. Retention time was observed at 1.64, mass spectroscopy method for βHB was set for detecting 102.9/58.8 (m/z). MultiQuant (Ab Sciex) was used to quantify the peaks against 6-point standard curve. Micromolar concentrations were normalized to the protein content.

### RNA seq

Total RNA was extracted from frozen liver tissue from 8- to 10-week-old, chow-fed, 24-h-fasted male control, Tsc1^L−/−^, Cpt2^L−/−^, and Tsc1Cpt2^L−/−^ mice using TRIzol reagent, further purified with RNeasy Mini Kit (QIAGEN) as directed by the manufacturer. RNA quality was assessed, and RNA samples were subjected to downstream analysis as described previously (12).

### Statistical analysis

Data were analyzed with Prism. Heatmap and PCA were generated by MetaboAnalyst (https://www.metaboanalyst.ca). Significance was determined using one-way ANOVA or two-way ANOVA with Tukey’s post hoc correction for multiple variable experiments.

## Data availability

RNA-seq data have been deposited in Gene Expression Omnibus GSE165701.

## Conflict of interest

The authors declare that they have no conflicts of interest with the contents of this article.
